# Resveratrol attenuates hypoxia-induced neuronal cell death, inflammation and mitochondrial oxidative stress by modulation of TRPM2 channel

**DOI:** 10.1038/s41598-020-63577-5

**Published:** 2020-04-15

**Authors:** Yener Akyuva, Mustafa Nazıroğlu

**Affiliations:** 1 Departmant of Neurosurgery, Faculty of Medicine, Hatay Mustafa Kemal University, Hatay, Turkey; 2 Director of Neuroscience Research Center (NOROBAM), Suleyman Demirel University, Isparta, Turkey; 3 Drug Discovery Unit, BSN Health, Analysis and Innovation Ltd. Inc. Teknokent, Isparta, Turkey

**Keywords:** Ion channels in the nervous system, Hypoxic-ischaemic encephalopathy

## Abstract

Hypoxia (HYPX) induced-overload Ca^2+^ entry results in increase of mitochondrial oxidative stress, inflammation and apoptosis in several neurons. Ca^2+^ permeable TRPM2 channel was gated by ADP-ribose (ADPR) and reactive oxygen species (ROS), although its activity was modulated in HYPX-exposed neurons by resveratrol (RSV). The aim of this study was to evaluate if a therapy of RSV can modulate the effect of HYPX in the TRPM2 expressing SH-SY5Y neuronal and HEK293 (no expression of TRPM2) cell lines. The SH-SY5Y and HEK293 cells were divided into four groups as control, RSV (50 μM and 24 hours), and HYPX and RSV + HYPX. For induction of HYPX in the cells, CoCl_2_ (200 μM and 24 hours) incubation was used. HYPX-induced intracellular Ca^2+^ responses to TRPM2 activation were increased in the SH-SY5Y cells but not in the HEK293 cells from coming H_2_O_2_ and ADPR. RSV treatment improved intracellular Ca^2+^ responses, mitochondrial function, suppressed the generation of cytokine (IL-1β and TNF-α), cytosolic and mitochondrial ROS in the SH-SY5Y cells. Intracellular free Zn^2+^, apoptosis, cell death, PARP-1, TRPM2 expression, caspase −3 and −9 levels are increased through activating TRPM2 in the SH-SY5Y cells exposed to the HYPX. However, the values were decreased in the cells by RSV and TRPM2 blockers (ACA and 2-APB). In SH-SY5Y neuronal cells exposed to HYPX conditions, the neuroprotective effects of RSV were shown to be exerted via modulation of oxidative stress, inflammation, apoptosis and death through modulation of TRPM2 channel. RSV could be used as an effective agent in the treatment of neurodegeneration exposure to HYPX.

## Introduction

Extensive death in neurons was induced by acute hypoxia, because disability and mortality of the neurons were increased by acute hypoxia^1^. Low blood flow to the tissue and low oxygen content of blood result in hypoxia and ischemic condition^2^. Cell survival decreased in the absence of oxygen, because ATP generation requires oxygen consumption in mitochondria^3^. Mitochondria is a main source of reactive oxygen species (ROS) generation^4^. Accumulating evidence indicates that the hypoxia and ischemic conditions result in excessive ROS generation, inflammation and apoptosis through the increase of membrane depolarization in mitochondria of neurons^5,6^. The increase of mitochondrial membrane depolarization was induced by the increase of intracellular free Ca^2+^ ([Ca^2+^]_i_) concentration. Recently, hypoxia-induced mitochondria ROS generation was inhibited through modulation of voltage gated calcium channel (VGCC) in the heart cells by resveratrol (RSV) treatment^7,8^. Hence, RSV can be useful for treatment of hypoxia in neuronal cells by modulation of mitochondrial ROS generation and the subject should be clarified in the hypoxia-induced SH-SY5Y neuronal cells.

Several neuronal physiological functions such as mitochondria and cell development are triggered by the changes of the [Ca^2+^]_i_ concentration^4^. In addition, several neurotoxicity functions such as apoptosis and inflammation in hypoxia are also induced by the increase of [Ca^2+^]_i_ concentration^9^. Hence, strict control of the [Ca^2+^]_i_ concentration through modulation of calcium channels is important for regulation of the physiologic and pathophysiologic conditions. In addition to the well-known calcium channels such as VGCC and ligand channels, members of transient receptor potential (TRP) superfamily with 28 members in mammalian cells were discovered within last decades^4^. Some members of the TRP superfamily such as TRP melastatin 2 (TRPM2) and TRP ankyrin 1 (TRPA1) are activated in several cells and neurons by ROS^10^. In addition to ROS, the TRPM2 is activated in several neurons such as dorsal root ganglion (DRG) and SH-SY5Y by ADP-ribose (ADPR), although it is blocked by antioxidants^11–13^. In SH-SY5Y cells, increase of [Ca^2+^]_i_ concentration through activation of TRPM2 channel induces increase the rate of caspase activation and apoptosis^14^. This pertains to neuronal cells, because TRP channels serve as targets for therapeutic agents that limit apoptosis^15^. Generation of hypoxia-inducible factors are high in the hypoxic conditions and they have major role in the adaptive responses to hypoxia^16^, but they are also activated by TRPA1 channel activation^16,17^. TRPM2 channel might be activated in SH-SY5Y neuronal cells by hypoxia-induced mitochondria ROS generation, although the subject still remains uninvestigated.

RSV (trans-3, 4′, 5-trihydroxystilbene) is a unique phytoalexin found in plants and fruits such as grapes and grape products. Its strong antioxidant action induced protective action against hypoxia-induced ROS generation and cytotoxicity in several neurons^18,19^. TRPM2 channel is activated in neurons by ADPR through ischemic-injury induced poly(ADPR) glycohydrolase (PARG)/poly ADP-ribose polymerase-1 (PARP-1) activation of the nucleus, although inhibition of PARP-1 activity induced TRPM2 blocker action^20^. Modulation of PARP-1 activity in microglia and SH-SY5Y neurons through RSV treatment was reported^21,22^. Modulation of experimental Parkinson’s disease-induced TRPM2 activation in the dopaminergic neuron of mouse via RSV treatment was also recently reported^14^. However, whether RSV acts a neuroprotective role against hypoxia-activated TRPM2 signaling pathways is still unknown.

There is no report the modulator action of RSV against the neurotoxicity via modulation of TRPM2 channel in the SH-SY5Y neurons following hypoxia. Hence, we propose that modulation of TRPM2 in the RSV treatment might represent a mechanism controlling adverse neurotoxicity actions in the SH-SY5Y neuronal cells with hypoxia. One of the well-known hypoxia mimetic agents through inhibition of cellular oxygen uptake in cell line models is cobalt chloride (CoCl_2_)^17,23,24^. It has been used in SH-SY5Y cell model for induction of hypoxia (HYPX). Hence, we used CoCl_2_ incubation in the SH-SY5Y model for induction of HYPX.

## Results

### Hydrogen peroxide induced TRPM2-dependent increase of Ca^2+^ fluorescence intensity in the SH-SY5Y cells overexpressing human TRPM2 channel cell but not in the HEK293 cells (in the absence of TRPM2)

**Figure 1:**
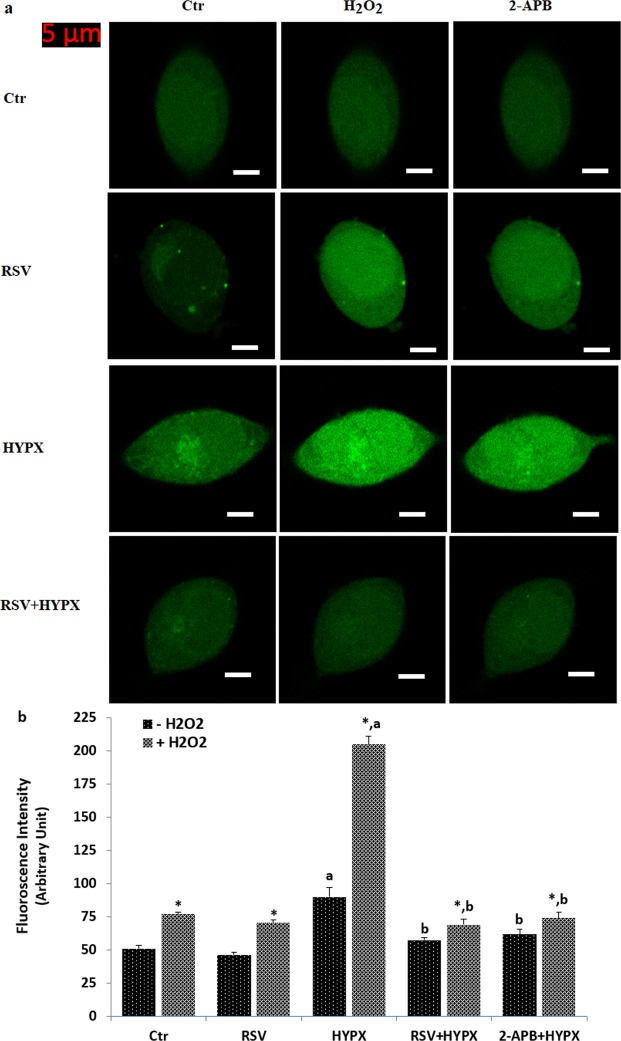
Effects of resveratrol (RSV and 50 μM) on the hypoxia (HYPX)-induced increase of [Ca^2+^]_i_ concentration in the SH-SY5Y cells (mean ± SD). The cells in the four groups were stained with Fluo-8 (1 μM for 30 min) and then the stained cells in the TRPM2 experiments were stimulated by H_2_O_2_ (1 mM for 10 min), but they were inhibited by 2-APB (100 μM for 5–10 min). Representative images of the effect of RSV and HYPX on the [Ca^2+^]_i_ levels through TRPM2 in the confocal microscope analyses were shown Fig. 1a. Changes of intensity of the [Ca^2+^]_i_ levels were shown by columns (Fig. 1b). The scale bar = 5 µm. Objective: 40x oil. One example of each figure was taken from 6 independent experiments, with each experiment examining 20 cells for each condition (^a^p ≤ 0.001 versus control (Ctr). ^b^p ≤ 0.001 versus HYPX group. ^*^p ≤ 0.001 versus - H_2_O_2_ group).

**Figure 2:**
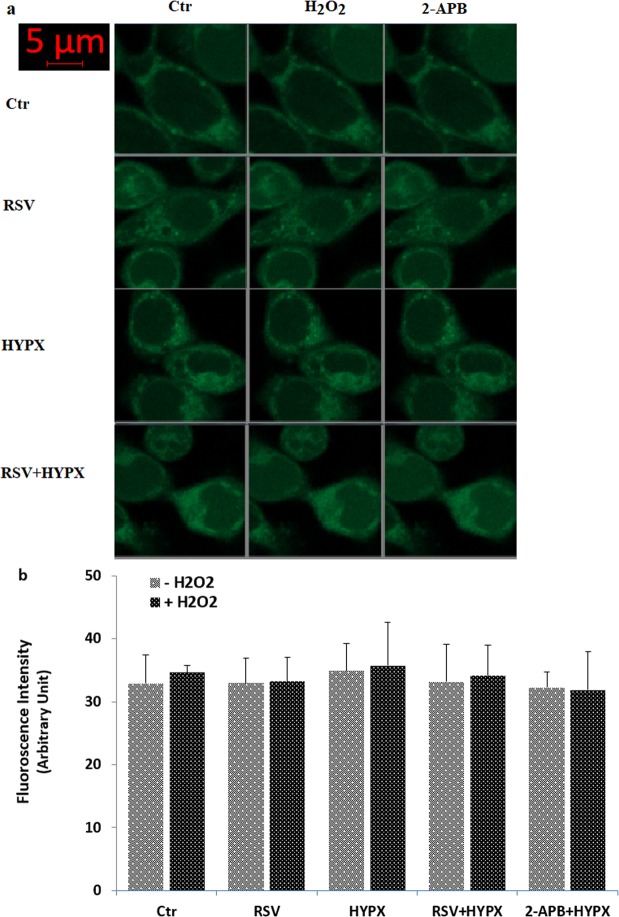
There was no changes on the [Ca^2+^]_i_ concentration in the HEK293 cells (in the absence of TRPM2) by hydrogen peroxide (H_2_O_2_). (mean ± SD). The cells were stained with Fluo-8 calcium dye and mean ± SD of fluorescence in 15 mm^2^ of the cells as arbitrary unit are presented; n = 20 independent experiments. The HEK293 cells were stimulated by H_2_O_2_ (1 mM for 10 min), but they were inhibited by 2-APB (100 μM for 10 min). The samples were analyzed by the LSC microscopy fitted with a 40× oil objective. The scale bar was 5 µm. Representative images (a) and column (b) of fluorescence intensities of the H_2_O_2_ and 2-APB on the TRPM2 activation in the LSC microscope analyses are shown in Figs a and b, respectively.

As the first step in the current study whether activations of TRPM2 are related to HYPX treatment, the influences of the channels on [Ca^2+^]_i_ concentration was examined by measurement of [Ca^2+^]_i_ concentration using the TRPM2 channel activator (H_2_O_2_) and inhibitors [2-aminoethyl diphenylborinate, 2APB and N-(p-amylcinnamoyl) anthranilic acid, ACA]. In the results of the laser scan confocal (LSC) microscope images (Fig. 1a) and columns (Fig. 1b), the fluorescence intensity of [Ca^2+^]_i_ concentration was increased in HYPX groups with or without H_2_O_2_ stimulation (via activation of TRPM2) (p ≤ 0.001). The fluorescence intensity of [Ca^2+^]_i_ concentration was significantly (p ≤ 0.001) lower in the RSV + HYPX and 2-APB + HYPX groups as compared to HYPX only. It is well known that there is no a functional TRPM2 expression in HEK293 cells without DNA transfection. Hence, we used HEK293 cells as a negative control of TRPM2 channel in the current study. There was no change in the results of the LSC microscope images (Fig. 2a) and columns (Fig. 2b) of [Ca^2+^]_i_ fluorescence intensity concentration in the four groups of HEK293 cells (in the absence of functional TRPM2 expression).

### RSV treatment diminished HYPX-induced increase of TRPM2 current densities in the SH-SY5Y cells but not in the HEK293 cells (in the absence of TRPM2)

**Figure 3:**
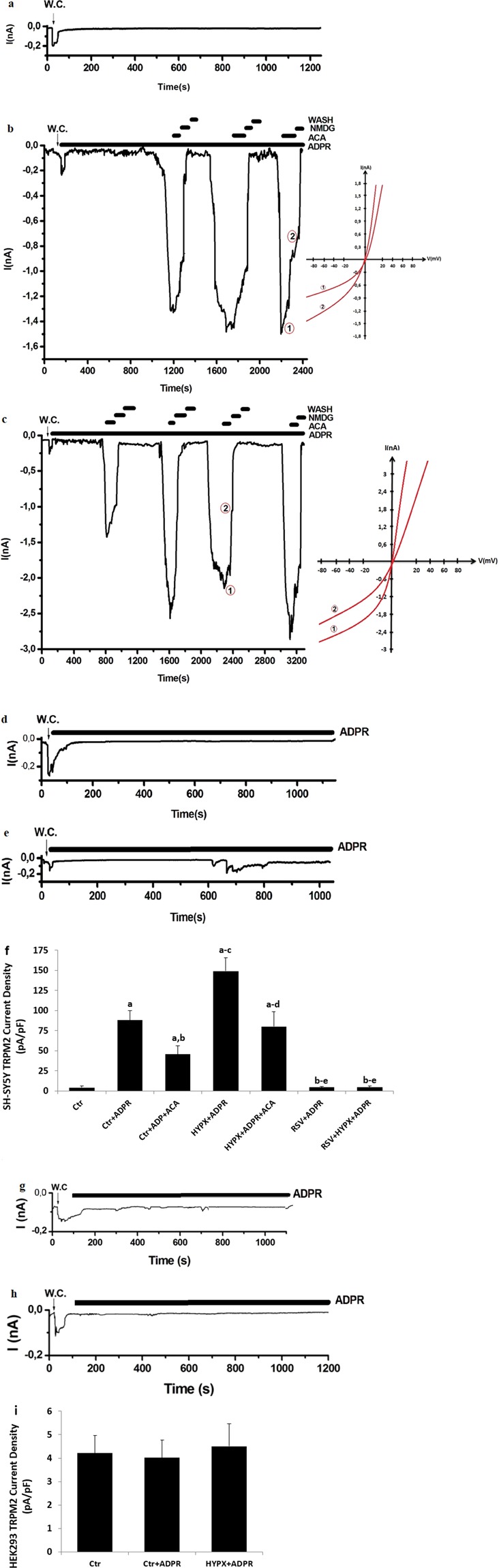
Hypoxia (HYPX)-induced the TRPM2 current densities (pA/pF) in the SH-SY5Y cells but not in the HEK293 cells were reduced by the resveratrol (RSV) treatment. (mean ± SD and n = 4–6). The TRPM2 currents in the SH-SY5Y cells were induced by intracellular ADPR (1 mM in the patch-pipette), but they were blocked by extracellular ACA (25 μM) in the patch-chamber. W.C. is abbreviation of whole cell record configuration. (**a**) Ctr (without ADPR stimulation): Original recordings from Ctr neuron. (**b**) ADPR group of control neuron (with ADPR stimulation). (**c**) HYPOX group. (**d**) RSV group. (**e**) RSV + HYPX group. The Fig. (**f**) was currents densities of a, b, c, d and e patch clamp records. (**g**) ADPR group of control HEK293 (with ADPR stimulation). (**h**) HYPOX group of HEK293 cells. The Fig. (**i**) was currents densities of g and h patch clamp records. (^a^p ≤ 0.001 versus control (Ctr). ^b^p ≤ 0.001 versus Ctr+ADPR groups. ^c^p ≤ 0.001 versus Ctr+ADPR + ACA group. ^d^p ≤ 0.001 versus HYPX + ADPR group. ^e^p ≤ 0.001 versus HYPX + ADPR + ACA group).

There were no currents in the absence of the intracellular ADPR (Fig. 3a) in the SH-SY5Y cells. We observed ADPR-induced increase of current density of TRPM2 channel in the patch-clamp experiments in the SH-SY5Y (Fig. 3b). However, the current densities were reversibly blocked by TRPM2 channel blocker (ACA) and NMDG^+^ (replacement of Na^+^). The current densities in the cells were significantly higher in the control+ADPR group (80.01 pA/pF) compared with the Control group (4.41 pA/pF) (p ≤ 0.001) (Fig. 3b); however, the current density in the SH-SY5Y neurons was lower in the control+ADPR + ACA group (45.54 pA/pF) as compared to the Control+ADPR group (80.01 pA/pF) (Fig. 2c,f) (p ≤ 0.001). The current densities in the SH-SY5Y neuronal cells were increased up to in 149.01 pA/pF in the HYPX group (Fig. 2d). There was no current and TRPV1 activation in the RSV (4.57 pA/pF) and RSV + HYPX (4.65 pA/pF) groups in the SH-SY5Y by the ADPR stimulation and they were significantly (p ≤ 0.001) low in the RSV + ADPR and RSV + HYPX + ADPR groups (Figs. 3d–f). In addition to patch-clamp analyses in the presence functional expression of TRPM2, we performed the patch-clamp experiments in the control, control+ADPR (Fig. 3g) and HYPX + ADPR (Fig. 3h) groups of HEK293 cells (in the presence of no functional TRPM2 expression). There was no statistical difference in the control (4.21 pA/pF), control+ADPR (4.01 pA/pF) and HYPX + ADPR (4.50 pA/pF) in the HEK293 cells. The present electrophysiology data indicated involvement of TRPM2 channel in the HYPX-induced excessive Ca^2+^ entry in the SH-SY5Y cells. However, the HYPX-induced TRPM2 currents were modulated in the cells by the antioxidant property of RSV treatment.

### RSV treatment modulated HYPX-induced cell viability (MTT), apoptosis, caspase −3, caspase −9, mitochondrial membrane depolarization (JC-1) and cytosolic ROS (DCFH-DA) generation changes in the SH-SY5Y neuronal cells

**Figure 4:**
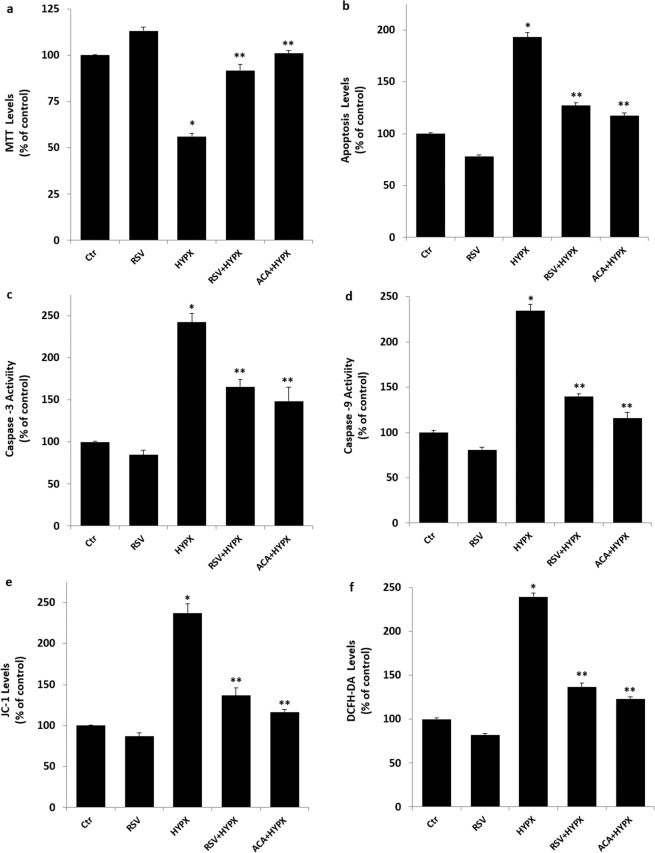
Resveratrol (RSV and 50 μM) and TRPM2 channel blocker (ACA and 25 μM) modulated hypoxia (HYPX)-induced cell viability (MTT), apoptosis, caspase -3, caspase -9, mitochondrial membrane depolarisation (JC-1) and cytosolic ROS generation (DCFH-DA) levels in the SH-SY5Y neuronal cells (mean ± SD and n = 3). The cell viability and apoptosis levels were analyzed in the spectrophotometer by using the MTT and commercial kit, respectively. However, caspase -3, caspase -9, mitochondrial membrane depolarization and cytosolic ROS generation in the SH-SY5Y neuronal cells were assayed in the microplate reader by using caspase -3 substrate (AC-DEVD-AMC), caspase -9 substrate (ACDEVD-AMC), JC-1 and DCFH-DA stains, respectively. (^*^p ≤ 0.001 versus control (Ctr) and RSV groups. ^**^p ≤ 0.001 versus HYPX group).

The electron transport system of mitochondria induces loss of mitochondrial membrane depolarization in the mitochondria resulting in excessive ROS generation and apoptosis via increases of caspase–3 and −9 activations^25,26^. For this reason, mitochondrial membrane depolarization is an important parameter of mitochondrial function and it was used as an indicator of ROS generation and apoptosis in neurons. The results of plate reader analyses of MTT (Fig. 4a), apoptosis (Fig. 4b), caspase −3 (Fig. 4c), caspase −9 (Fig. 4d), JC-1 (Fig. 4e) and DCFH-DA (Fig. 4f) levels are shown in Fig. 4. The apoptosis, caspase −3, caspase −9, JC-1 and DCFH-DA levels through activation of TRPM2 in SH-SY5Y were higher in the HYPX group than in the control and RSV groups, although MTT levels were lower in the HYPX group than in the control and RSV groups (p ≤ 0.001). However, the apoptosis, caspase −3, caspase −9, JC-1 and DCFH-DA levels were lower in the RSV + HYPX and ACA + HYPX groups as compared to HYPX group, although MTT level was increased in the RSV + HYPX and ACA + HYPX groups by the RSV and ACA treatments (p ≤ 0.001).

### RSV and 2-APB diminished increase of fluorescence intensities of mitochondrial membrane depolarization (JC-1), cytosolic (DCFH-DA and DHR123) and mitochondrial (MitoROS) ROS generation in the SH-SY5Y neuronal cells

**Figure 5:**
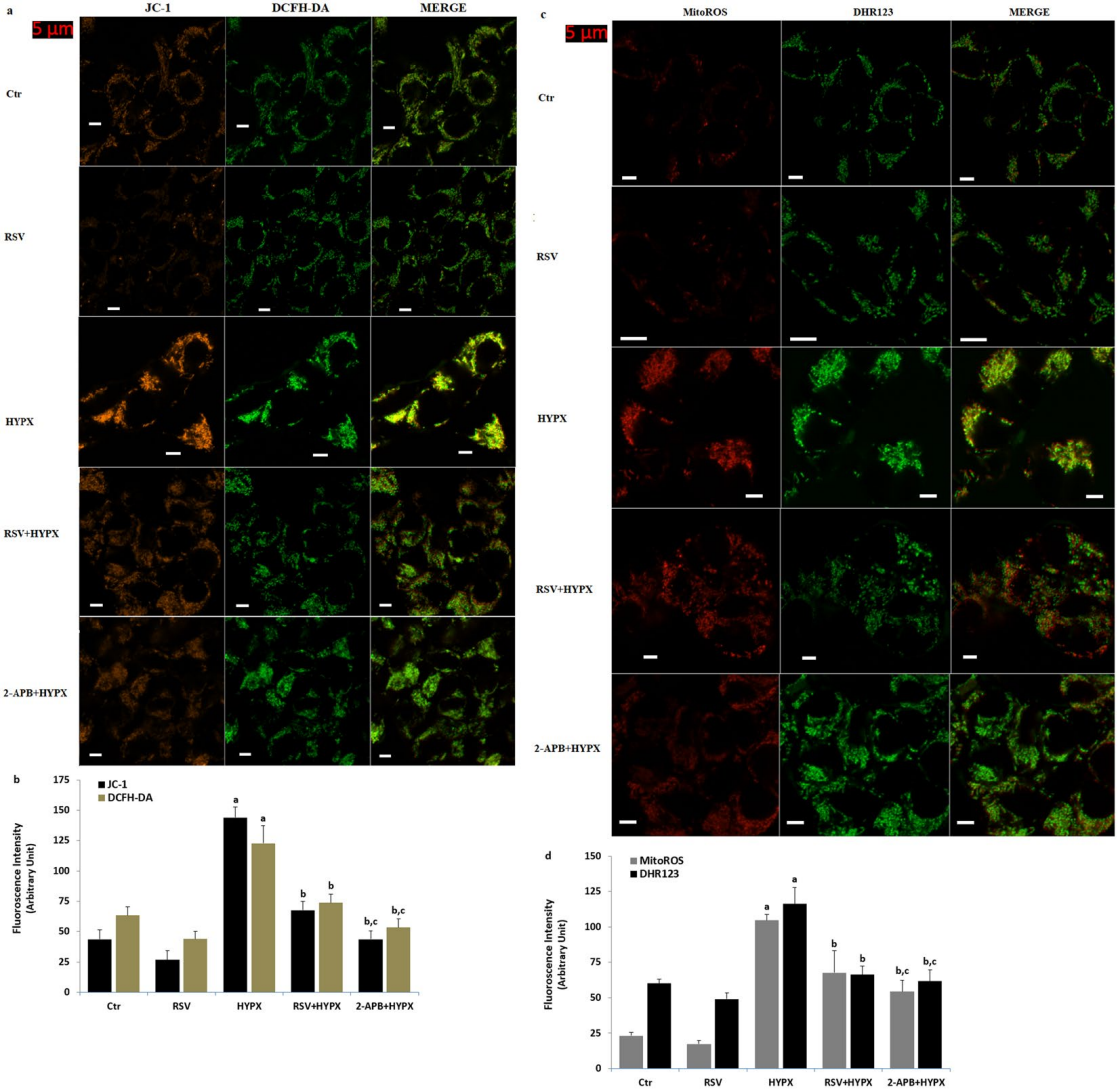
Effect of resveratrol (RSV and 50 μM) and 2-APB (100 μM) on the mitochondrial membrane depolarization (JC-1), cytosolic (DHR123 and DCFH-DA) and mitochondria (MitoROS) ROS generation levels in the hypoxia (HYPX)-induced SH-SY5Y cells. Mean ± SD of fluorescence in 15 mm^2^ of the cells as arbitrary unit are presented; n = 20 independent experiments. The SH-SY5Y neuronal cells were stimulated by H_2_O_2_ (1 mM for 10 min), but they were inhibited by 2-APB (100 μM for 10 min). The samples were analyzed by the LSC microscopy fitted with a 40× oil objective. The scale bar was 5 µm. Representative images and column of fluorescence intensities of the JC-1 + DHR123 (Fig. 5a,b) and DCFH-DA + MitoROS (Fig. 5c,d) on the TRPM2 activation in the LSC microscope analyses are shown in Fig. 5a–d, respectively. (^a^p ≤ 0.001 versus control (Ctr) and RSV groups. ^b^p ≤ 0.001 versus HYPX group. ^c^p ≤ 0.001 versus RSV + HYPX group).

In addition to the microplate reader analyses of mitochondrial membrane depolarization (JC-1) and cytosolic (DCFH-DA) and mitochondria (MitoROS) ROS generation in the SH-SY5Y, we want to further confirm the changes in the cells by using LSC microscope analyses. Compared with control, HYPX (CoCl_2_) treatment increased the fluorescence intensity of JC-1 (Fig. 5a,b), DCFH-DA (Fig. 5a,b), MitoROS (Fig. 5c,d) and DHR123 via activation of TRPM2 (Fig. 5c,d) (p ≤ 0.001). Importantly, we found RSV and 2-APB reduced the fluorescence intensities of the JC-1, DCFH-DA, MitoROS and DHR123 in the cells (p ≤ 0.001). Our data further suggested that CoCl_2_**-**induced mitochondria membrane depolarization, cytosolic and mitochondrial ROS generation, which could be modulated by RSV treatment through inhibition of TRPM2 channel.

### HYPX-induced SH-SY5Y neuronal death was diminished by the RSV and 2-APB treatments

**Figure 6:**
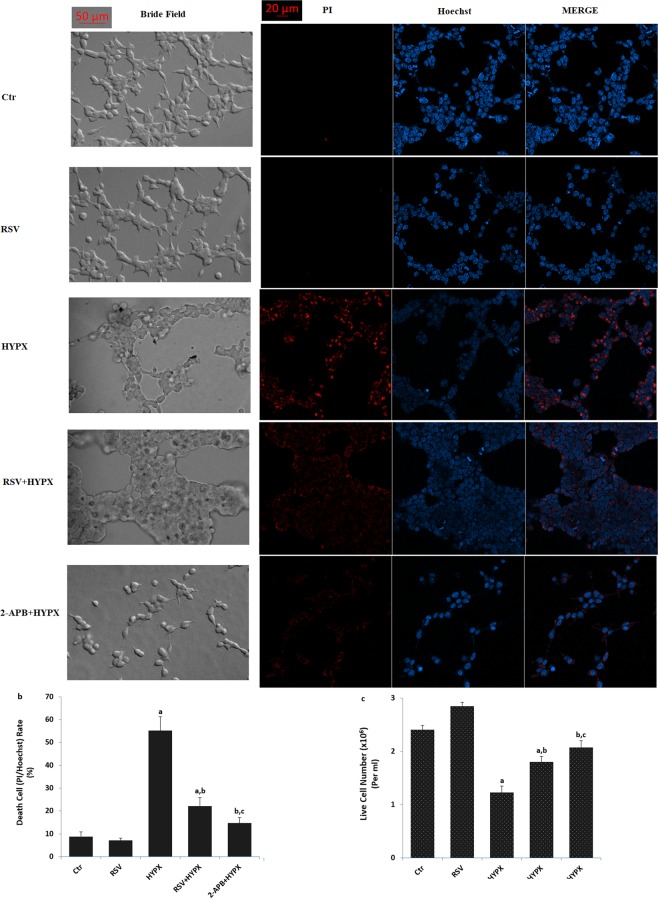
Resveratrol (RSV and 50 μM) and 2-APB (100 μM) protected hypoxia (CoCl_2_ and 50 μM)-induced cell death and cell number changes in the SH-SY5Y cells. (mean ± SD). (**a**) Each panel consists of PI (red) and Hoechst (blue)-staining, and bride field (black-white) images are showing dead and live cells. The scale bar is 50–20 μm. (**b**) Summary of the mean percentage of PI and Hoechst-positive cells under the indicated conditions from 6 independent experiments, with each experiment examining 20–25 cells for each condition. (**c)** Mean numbers of the cell count (n = 6). (^a^p ≤ 0.001 versus control (Ctr) and RSV groups. ^b^p ≤ 0.001 versus HYPX group. ^c^p ≤ 0.001 versus RSV + HYPX group).

Accumulating evidences suggest that HYPX induced death and apoptosis is mainly through the increase of TRPM2 activation in the neurons such as DBTRG glioblastoma and hippocampus^10,24^. However, there is no report on the neurotoxicity and neuronal death in the CoCl_2_-treated SH-SY5Y neurons. After observing the increase in cytosolic and mitochondrial ROS through TRPM2 activation in the SH-SY5Y neuronal cells, we suspected whether cell number and cell death was changed in the neuronal cells. The percentage of dead cells was markedly (p ≤ 0.001) higher in the HYPX group than in the control and RSV groups (Fig. 6a,b), although cell number was decreased in the group (Fig. 6c). However, RSV and 2-APB induced cell protective actions against the cell death and the percentage of dead cells was lower in the HYPX + RSV and HYPX + 2-APB groups as compared the HYPX group, although cell number counts were increased in the groups by the RSV and 2-APB treatment (p ≤ 0.001).

### HYPX-induced fluorescence intensity of free zinc ion (Zn2^+^) in the SH-SY5Y neuronal cells was diminished by the RSV and 2-APB treatments

**Figure 7:**
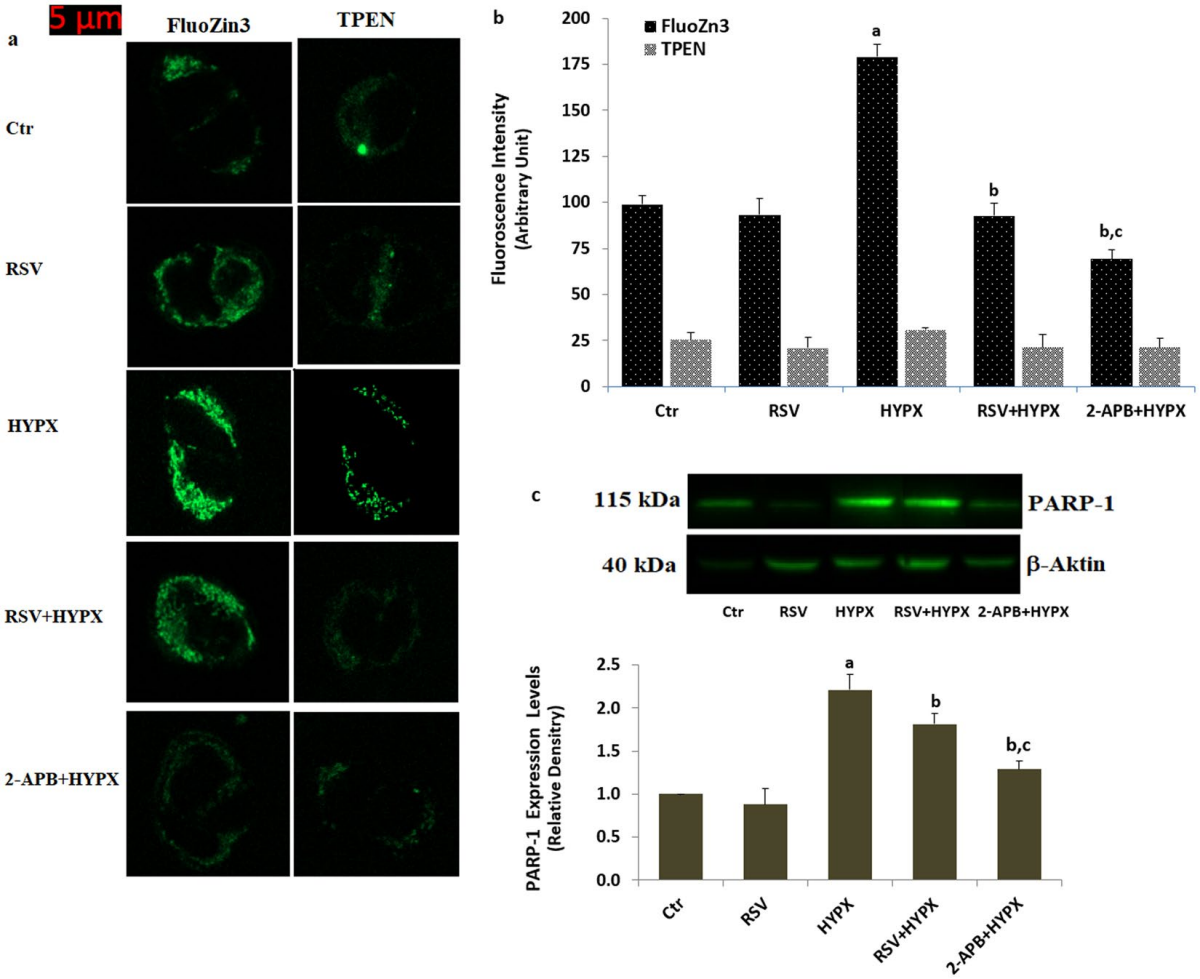
Resveratrol (RSV and 50 μM) and 2-APB (100 μM) protected hypoxia (CoCl_2_ and 50 μM)-induced increase of intracellular free Zn^2+^ concentration and PARP-1 expression level in the SH-SY5Y cells. (mean ± SD). (**a**) Each panel consists of FluoZn3 and TPEN-staining images are showing presence and absence of intracellular free Zn^2+^, respectively. The scale bar is 5 μm. (**b**) Summary of the mean percentage of FluoZn3 and TPEN-positive cells under the indicated conditions from 6 independent experiments, with each experiment examining 20–25 cells for each condition. (**c**) PARP-1 expression level. (^a^p ≤ 0.001 versus control (Ctr) and RSV groups. ^b^p ≤ 0.001 versus HYPX group. ^c^p ≤ 0.001 versus RSV + HYPX group).

Accumulating evidence indicated that an increase of intracellular free Zn^2+^ concentration induces excessive ROS generation^27,28^. After the finding observable increase in the intracellular ROS generation in the SH-SY5Y cells, we suspected increase of the intracellular free Zn^2+^ concentration. Hence, we decided to measure intracellular free Zn^2+^ concentration in the LSC microscope by using a fluorescent dye (FluoZin3). The fluorescence intensity of FluoZin3 in the HYPX group was increased as compared to the control and RSV groups (p ≤ 0.001) (Fig. 7a,b). However, its fluorescence intensity was decreased in the RSV + HYPX and 2-APB + HYPX groups by the RSV and 2-APB treatments (p ≤ 0.001). TPEN is an inhibitor of the intracellular free Zn^2+^ concentration^27^. After TPEN treatment (100 nM) as a selective Zn^2+^ chelator, almost fully protected HYPX-induced oxidative neurotoxicity, indicating a vital role of a rising intracellular free Zn^2+^ concentration in HYPX-induced neurotoxicity (Fig. 7a,b). These imaging results confirmed the protective role of RSV on the HYPX-induced free Zn^2+^ accumulation in the SH-SY5Y cells.

### HYPX-induced PARP-1 expression, TRPM2 mRNA and cytokine levels were diminished in the SH-SY5Y neuronal cells by the RSV treatment

TRPM2 channel is activated by increasing PARP-1 activation^20,29^. In addition, TRPM2 channel expression and cytokine generation levels are increased by the HYPX. After observing increase of Ca^2+^ influx via activation of the TRPM2 channel in the cells, we suspected increased expression levels of PARP-1 and TRPM2, and generations of IL-1β and TNF-α in the cells. The PARP-1 expression level is shown in the Fig. 7c and Supplementary Fig. 1, although TRPM2 mRNA expression, IL-1β and TNF-α release levels are shown in the Supplementary Fig. 2. Accumulating evidence indicates that SH-SY5Y cell has a PARP-1 activation^28,30^. The PARP-1 expression, TRPM2 mRNA expression, IL-1β and TNF-α release levels were higher in the SH-SY5Y as compared to the control and RSV (p ≤ 0.001). The PARP-1 and TRPM2 mRNA results confirmed increased TRPM2 channel activation results in the SH-SY5Y cells by HYPX. However, the TRPM2 mRNA level, PARP-1, IL-1β and TNF-α activities in the cells were lower in the RSV + HYPX group than in the HYPX group (p ≤ 0.001). the PARP-1 activity in the cells was further decreased by the 2-APB treatment and its expression level were lower in the 2-APB + HYPX than in HYPX and RSV + HYPX groups. The results confirmed inflammatory property of HYPX via activation of PARP-1 and TRPM2 activation in the SH-SY5Y cells.

### Lipid peroxidation (MDA), rGSH level and GPx results

MDA, rGSH concentration and GPx activity results are shown in Table 1. The rGSH concentration and GPx activity were lower in the HYPX group than in the control and RSV groups, although the MDA concentration was high in the HYPX group (p ≤ 0.001). However, the GPx activity and rGSH concentration were increased in the HYPX + RSV and HYPX + ACA groups by the RSV and ACA treatments, although MDA concentration was decreased in the groups (p ≤ 0.001). These results clearly indicated that HYPX induced increase of MDA concentration is reduced in the SH-SY5Y neuronal cells by RSV and ACA treatments through upregulation of rGSH level and GPx activity.

**Table 1:** Effect of HYPX (CoCl_2_ and 50 μM), resveratrol (50 μM) and ACA (25 μM) on lipid peroxidation (MDA), reduced glutathione (rGSH) level and glutathione peroxidase (GPx) activity in the SH-SY5Y neuronal cells (mean ± SD and n = 6).

Values	Control	RSV	HYPX	RSV + HYPX	ACA + HYPX
MDA (μmol/ g protein)	22.90 ± 2.75	21.80 ± 1.90	25.20 ± 2.03*	20.00 ± 1.04^**^	17.40 ± 0.58^**^
rGSH (μmol/ g protein)	10.90 ± 0.30	12.20 ± 1.36	8.30 ± 0.48*	10.90 ± 0.45^**^	12.00 ± 0.49^**^
GPx (IU/ g protein)	17.40 ± 0.47	18.10 ± 2.12	12.00 ± 0.50*	18.40 ± 0.49^**^	23.20 ± 2.03^**^

^*^p ≤ 0.001 versus control and RSV groups. ^**^p ≤ 0.001 versus HYPX group. The data were analyzed by Student’s t test.

## Discussion

There are currently no effective protective agents against the injury action of HYPX. Recently, RSV as a natural product has attracted attention as a potential therapeutic agent based on its potential antioxidant or anti-apoptotic effects^3,6^. In the present study, we found that RSV incubation ameliorated HYPX-induced deleterious effects via modulating the intracellular Ca^2+^ hemostasis, inflammation, cell death, cell viability and apoptosis pathways, as well as down-regulating the PARP-1 expression level, mitochondrial and cytosolic oxidative stress pathways. The major findings of the study are that TRPM2 channels are activated in the SH-SY5Y (in the presence of functional expression of TRPM2) cells but not in HEK293 (in the absence of functional expression of TRPM2) cells by HYPX and its sensitivity enhance to ROS. However, RSV modulated TRPM2 activation through the upregulation of rGSH concentration and GPx activity and down-regulation of intracellular and mitochondrial ROS generation (Supplementary Fig. 3). Based on our results, we propose that the beneficial effects of RSV on the TRPM2 activation were based on promoting rGSH concentration and GPx activity and reducing mitochondrial and cytosolic oxidative stress, and restoring PARP-1 expression in the SH-SY5Y cells.

TRPM2 channel was first identified oxidative stress sensitive channel in the history of the TRP superfamily, because it has an oxidative stress sensitive enzyme namely ADPR pyrophosphatase in the C-terminal NUDT9 homology domain^20^. ROS also induce activation of the TRPM2 channel via promoting ADPR generation catalyzed by PARP-1 and PARG in the nucleus^20,29^. Consistent with the reports, we observed Ca^2+^ influx through the TRPM2 channel activation in the TRPM2 channel expressing SH-SY5Y neuronal cells but not in HEK293 (in the absence of TRPM2) cells by the H_2_O_2_ and ADPR stimulations. Ischemia/reperfusion-induced ROS was diminished via upregulation of antioxidant enzymes, but down-regulation of TRPM2 channel activity in cardiomyocytes of mice^31^. TRPM2 is activated in DRG and hippocampus of rats by depletion of rGSH and GPx, although treatment with rGSH acted TRPM2 channel blocker action through inhibition of PARP-1 activity in the neurons^32–34^. In the present study, we observed modulation of the TRPM2 channel via inhibition excessive ROS generation and increase of thiol redox cycle antioxidants (rGSH and GPx) in the HYPX-induced SH-SY5Y cell by the RSV and TRPM2 channel blocker (ACA and 2-APB) treatments. Similarly, we recently observed a modulator action of RSV on the TRPM2 channel activity in the DBTRG glioblastoma cells^24^.

In the current study, we observed increased levels of apoptosis, mitochondrial membrane depolarization and ROS via stimulation of TRPM2 in the SH-SY5Y cells by HYPX induction, although the levels were decreased in the cells by the RSV treatment. Mitochondria act major actions in neuron metabolism, energy homeostasis, the oxidative stress, apoptosis and cell death^4,35^. Regulation of mitochondrial Ca^2+^ uptake could counteract HYPX-induced mitochondrial membrane depolarization suppression, stimulate recovery of mitochondrial homeostasis, reduce neuron oxidative injury and apoptosis, and enhance tissue repair and regeneration in the SH-SY5Y neuronal cells^36,37^. The maintenance of mitochondrial membrane structure and physiological function depends on efficient mitochondrial function, which is restricted under hypoxic conditions^38^. It was reported that RSV modulates mitochondrial biogenesis and attenuates mitochondrial dysfunction, apoptosis and cell death in the rotenone-induced oxidative stress SH-SY5Y cellular Parkinson disease model^39,40^. In the present literature data, there is no report on the protective action of RSV against TRPM2 activation in cells during the HYPX. However, the results of our previous study indicated that RSV modulates mitochondrial biogenesis and apoptosis in DBTRG neuroblastoma cells via upregulation of TRPM2 channel activation and ROS generation during HYPX^24^. We found that RSV treatment of the SH-SY5Y neuronal cells subjected to CoCl_2_-induced HYPX modulated the structural membrane depolarization integrity and enhanced biogenesis of mitochondria and restored mitochondrial and cytosolic ROS generation functions via modulation of TRPM2. We examined whether TRPM2 channel blockers (ACA and 2-APB) mediates the effects of HYPX and RSV on mitochondria, since results of recent studies have shown that ROS generation and Ca^2+^ influx via VGCC activation is important for the mitochondrial response to stress and may be linked to neuronal apoptosis and death caused by HYPX^5,8^. Consistent with present findings, the mitochondria membrane depolarization, ROS generation, apoptosis and cell via stimulation of [Ca^2+^]_i_ increase and TRPM2 channel activation were upregulated in the SH-SY5Y cells but not in the HEK293 cells exposed to HYPX; importantly, these were totally reversed by RSV and TRPM2 channel blocker (ACA and 2-APB) treatments. We also found that PARP-1 activation in the cultured SH-SY5Y cells subjected to HYPX was decreased by RSV treatment. These data indicate that RSV may preserve SH-SY5Y neuronal function by modulating mitochondrial homeostasis via [Ca^2+^]_i_ concentration and TRPM2 channel activation.

Accumulating evidence indicates that the injuring effects of HYPX are exerted at least in part by excessive ROS and cytokine generation^16,19,38^. We observed increased ROS, IL-1β and TNF-α generations and MDA levels in the SH-SY5Y cells exposed to HYPX, which were diminished by RSV treatment. However, we observed decrease of rGSH level and GPx activity in the SH-SY5Y cells exposed to HYPX, which were upregulated by the RSV treatment. These data are agreement with previous results showing decreased oxidation of thiol redox system members in the SH-SY5Y cells after RSV treatment, providing evidence for a protective effect of RSV through upregulation of rGSH and GPx in diminishing neuronal oxidative stress and inflammation during HYPX^3,24^. The observed decreases in ROS and MDA upon exposure of HYPX may therefore be due to the increase repair of respiratory chain enzyme complex activity through upregulation of rGSH and GPx redox system in the mitochondria of SH-SY5Y cells. Consistent with these findings, treatment of RSV diminished mitochondrial ROS and MDA through upregulation of rGSH and GPx in the brain of neonatal rats with hypoxic-ischemic encephalopathy^41^ and the HYPX/reoxygenation-induced H9c2 cardiomyocyte cell line^42^.

Neuronal apoptosis through activations of caspase −3 and −9 have been implicated in HYPX-induced TRPM2 channel activations^24^. The present study confirmed that oxidative neurodegeneration-induced death in the SH-SY5Y cells was increased by HYPX induction. RSV treatment increased the levels of cell viability (MTT) and protected neuronal cell count. Consistent with these observations, several studies have found that antioxidants such as curcumin neuroprotective actions through inhibition of TRPM2 channel and caspase activations against apoptosis and neuronal cell death under hypoxic conditions^38,43^. In the results of a recent study, RSV was shown to improve oxygen glucose deprivation-induced ischemic stroke via modulation mitochondrial dysfunction in the SSY5Y cells^44^. Consistent with the literature results, we observed modulator role of RSV on the apoptosis and neuronal cell death via inhibition of caspase −3 and −9 activities in the SH-SY5Y neuronal cells.

Zn^2+^ as a component of some antioxidant enzymes contributes to reducing excessive ROS generation in several cells and neurons^38^. Free Zn^2+^ has apoptosis and oxidant actions via accumulation in the mitochondria for neurons^27^. Hence, intracellular free Zn^2+^ depletion causes decrease in mitochondria ROS generation, caspase activities and [Ca^2+^]_i_ concentrations via increase of rGSH and GPx antioxidant levels in the renal collecting duct cells^45^. It is well known that grape products contain rich amount of RSV and hepatoprotective effect of grape seed oil against carbon tetrachloride-induced oxidative stress, Zn^2+^ accumulation, rGSH depletion, apoptosis, caspase-3 activation in radiation exposed rats were recently reported^46^. In the current study, we indicated that HYPOX-induced intracellular free Zn^2+^ and Ca^2+^ increases resulted in increases of mitochondrial membrane depolarization which is associated with an increase in the excessive Ca^2+^ influx and mitochondria ROS. We observed that the intracellular free Zn^2+^ increase in the SH-SY5Y cells was diminished by the RESV and 2-APB treatments. Hence, the treatments lead to decrease of ROS-mediated caspase, mitochondrial dysfunction and TRPM2 activation resulting in a decrease of apoptosis and cell death.

In summary, the present study provides the first evidence that RSV treatment in the TRPM2 channel expressing SH-SY5Y neuronal cells but not HEK293 cells can ameliorate the mitochondria oxidative stress, caspase −3, caspase −9, apoptosis and cell death induced by HYPX by improving TRPM2 channel activation, decreasing mitochondrial oxidative stress, and protecting rGSH and GPx thiol redox system. RSV treatment also diminished cell death and cell count by reducing the ROS generation in the SH-SY5Y neuronal cells (Supplementary Fig. 3). These effects resulted from an increase in the restoration of mitochondrial function via modulation of TRPM2 channel and calcium signaling. The current findings suggest a new treatment target via for preventing HYPX-induced neurodegeneration, TRPM2 channel activation and oxidative injury.

## Materials and methods

### Cell culture

In the current study, we used SH-SY5Y neuronal and HEK293 kidney cell lines and they were originally purchased from Ankara Şap Institute, Ministry of Food, Agriculture and Livestock, Republic of Turkey. The cells were cultured in a medium consisting of DMEM medium mixture as described in previous studies^13,29,43^. The cells were kept in a humidified atmosphere in 5% CO_2_ at 37 °C (NB-203QS, N-BIOTEK, Genbiotek Biosistem Inc., Buyukcekmece, Istanbul, Turkey). After counting the cell in an automatic cell counter (Casy Modell TT, Roche, Germany), they were seeded in 4 flasks at a density of 1 × 10^6^ cells per flask with filter cap.

### Groups

Naturel presence of TRPM2 channel in SH-SY5Y but not in the HEK293 cells were indicated by the expression studies^29,47,48^. The SH-SY5Y cells as neuronal disease models have been used in several studies^13,29,43^. Hence, the SH-SY5Y cells were preferred on the investigation of the channels in the present study, although the HEK293 cells were used absence of TRPM2 channel (negative control) in the current study. The SH-SY5Y and HEK293 cells were divided into four main groups as follows;

#### Control group

The cells were kept in under the same cell culture medium and conditions for 48 hours without TRPM2 channel blockers (ACA and 2-APB), RSV and CoCl_2_ treatments.

#### RSV group

After keeping 24 hours in the cell same culture condition without treatment, the cells in the group were pre-incubated with RSV (50 μM) for 24 hours as described in a previous study^23,24^.

#### Hypoxia (HYPX) group

After keeping 24 hours in the cell same culture condition, the cells were incubated with CoCl_2_ (200 μM) for 24 hours as described in previous studies^17,23,24^.

#### HYPX + RSV group

After pre-incubation with RSV (50 μM) for 24 hours, the cells in the group were incubated with CoCl_2_ (200 μM) for a further period of 24 hours.

The RSV, ACA, 2-APB, and ADPR were purchased from Santa Cruz Inc, (Istanbul, Turkey) and their stock solutions were prepared in dimethyl sulfoxide. After diluting in extracellular solution, and the pH adjustments of the ACA, 2-APB, ADPR and hydrogen peroxide (H_2_O_2_), they were freshly incubated with the cells.

### Measurement of intracellular Ca^2+^ fluorescence intensity through TRPM2 potentiation in the SH-SY5Y neurons in LSC microscope

We investigated RSV-induced TRPM2 potentiation in the SH-SY5Y neurons and HEK293 cells by LSC microscope (LSM800, Zeiss, Ankara, Turkey) analyses as described in previous studies^45,49^. The cells were seeded 35 mm glass bottom dishes (Mattek Corporation Inc., Ashland, MA, USA) before the analyses. Intracellular changes in the Ca^2+^ fluorescence intensity in the SH-SY5Y neurons were monitored by using 1 μM fluorescent dye (Fluo-8, Calbiochem, Darmstadt, Germany). The Fluo-8 is a single wavelength excitation and emission dye that excited by a 488 nm argon laser from the confocal microscope (LSM 800). The cells were treated with 2-APB (100 μM) to inhibit Ca^2+^ entry before stimulation of TRPM2 (H_2_O_2_ and 1 mM). The neurons were analyzed at 515 nm by the LSC microscopy fitted with a 40x oil objective by using ZEN program. The results of Fluo-8 were expressed as the mean fluorescence intensity as arbitrary units per cell.

### Electrophysiology

For taking whole-cell (W.C) configuration of patch-clamp records at room temperature (21 ± 1 °C), we used voltage clamp recording in the SH-SY5Y and HEK293 cells (EPC10 patch-clamp set, HEKA, Lamprecht, Germany). Except calcium concentration in the intracellular solution, we used standard extracellular bath and pipette solutions as described in previous studies^11,12,23^. Activation of TRPM2 channel in the presence of high intracellular Ca^2+^ concentration was reported^50^. Hence, we used 1 μM intracellular Ca^2+^ concentration instead of physiologic intracellular Ca^2+^ concentration (50–100 nM). For calculation of intracellular Ca^2+^ concentration in the intracellular and extracellular solutions, we used the MAXC-program (http://www.stanford.edu/,cpatton/maxc.html) as described in a previous study^29^. For obtaining Na^+^ free solution, we used N-methyl-D-glucamine (NMDG) instead of Na^+^. Titration of the NMDG solution was performed with HCl. Minus 60 mV in the cells was used as holding potential of the voltage clamp patch-clap analyses. Voltage clamp technique was used in the analyses and current-voltage (I-V) relationships were obtained from voltage ramps from −150 to +150 mV applied over 200 milliseconds. In the patch-clamp experiments, the TRPM2 channel was intracellularly (via intracellular solution of patch pipette) gated by ADPR (1 mM), and the channels were extracellularly blocked by ACA (25 μM). The maximal current amplitude (pA) in a SH-SY5Y or a HEK293 cell was divided by the cell capacitance (pF), a measure of the cell surface. Results of current density were expressed as pA/pF in the patch-clamp analyses.

### Microplate reader assay of cell viability (MTT)

Viability assays were performed by measuring mitochondrial reductase activity with MTT [3-(4,5-dimethylthiazol-2-yl)−2,5-diphenyltetrazolium bromide] (Sigma-Aldrich, Istanbul, Turkey) as described in previous studies^34,45,49^. Cells from the four groups were incubated in MTT solution (0.5 mg/ml) for 15 min and the media were removed. The resulting formazan crystals were dissolved in dimethyl sulfoxide (100 μl/well). Absorbance in a microplate reader (Infinite pro200; Tecan Inc, Groedig, Austria) was read at 550 nm. The data are presented as percentage (%)-increase over the pretreatment level.

### Microplate reader assay of apoptosis, caspase 3 and 9 activities

For detecting the apoptosis level, we used a commercial kit (Biocolor Ltd., Northern Ireland) and the analyses were performed in a spectrophotometer (UV-1800, Shimadzu, Kyoto, Japan). Details of the apoptosis analyses were given in instructions of Biocolor Ltd. and elsewhere^45,49^.

To determine caspase-3 and −9 activity, the neurons in the control and treated with ACA groups were incubated with 2 ml of substrate solution for 1 h at 37 °C as previously described^45,49^. For assay of caspase-3 and −9 activities, cleavages of fluorogenic substrates (AC-DEVD-AMC for caspase −3 and ACDEVD-AMC for caspase-9) (Bachem, Bubendorf, Switzerland) were used. The substrate cleavages were measured with the automatic microplate reader (Infinite pro200) with excitation wavelength of 360 nm and emission at 460 nm. After calculation of fluorescence units/mg protein, the data were presented as % of control.

### Measurement of intracellular reactive oxygen species (ROS) formation in the microplate reader and LSC microscope

2′,7′-Dichlorofluorescin Diacetate (DCFH-DA) is an oxidation-sensitive stain. It is a non-fluorescent compound, but it is converted to fluorescent form in the cytosol of cell, when it was taken up into cells. ROS formation was monitored using the microplate plate reader (Infinite pro200) described previously^25,26^. Briefly, the cells (1 × 10^6^) were incubated with 10 μM DCFH-DA at 37 °C for 30 min. The fluorescence increase, which is due to the hydrolysis of DCFH-DA to DCF by nonspecific cellular esterase and its subsequent oxidation by peroxides, was measured at 488 nm (excitation)/525 nm (emission) by the microplate plate reader (Infinite pro200).

Another oxidation-sensitive non fluorescent dye is dihydrorhodamine- 123 (DHR123). It is also a non-fluorescent compound, but the DHR123 is converted to fluorescent from (Rh123) in the cytosol of cell, when it was taken up into cells. The ROS formations via Rh123 and DCF were also imaged using the LSC microscopee (LSM800) as described previously^24^. Briefly, the neuronal cells (1 × 10^6^) were seeded in the glass bottom dishes and then they were incubated with 10 μM DHR123 at 37 °C for 30 min. Rh123 as a fluorescent form of DHR123 was excited with an Argon laser at 488 nm. Fluorescence intensity of each cells was monitored in the LSC microscope equipped with 40 × oil objectives. The data are presented as arbitrary unit.

### Measurement of mitochondrial membrane potential (ΔΨm) formation in the microplate reader and LSC microscope

5,5′,6,6′-Tetrachloro-1,1′,3,3′-tetraethylbenzimidazolylcarbocyanine iodide (JC-1, Molecular Probes, Eugene, OR, USA) accumulates in mitochondria according to ΔΨm level and is present either as monomer or reversible J-aggregate^25^. Details of the ΔΨm formation analyses were given in previous studies. Briefly, the cells (1 × 10^6^) in the microplate reader and LSC microscope analyses were incubated with 5 μM JC-1 at 37 °C for 30 min.

In the microplate JC-1 analyses, the green (excitation; 485 nm and emission; 535 nm) and red (excitation; 540 nm and emission; 590 nm) JC-1 signals were measured in the cell line as described in a previous study^25^. Fluorescence changes were analyzed using the microplate reader (Infinite Pro200). The data are presented as the fold-increase over the pretreatment level.

In the LSC microscope JC-1 analyses, the stain was excited with an Argon laser at 488 nm as described in previous studies^45,49^. Fluorescence intensity of each cells was monitored in the LSC microscope equipped with 40× oil objectives. Images were acquired with ZEN program and analyzed using Image J/Imaris software. The data are presented as arbitrary unit.

### Count of life cell number in the automatic cell counter and live (Hoechst)/ death (PI) analyses in the LSC microscope

As described in a previous study^25^, live cell number was counted in an automatic cell counter (Casy Modell TT). However, live and death rate of neuronal cells in the LSC microscope (LSM800) were determined by using Hoechst 33342 and propidium iodide (PI) staining, respectively. Briefly, the neuronal cells (1 × 10^6^) were seeded into the glass bottom dishes and then they were incubated with PI (5 μg/ml) and Hoechst 33342 (1 μM) (Cell Signaling Technology) at 37 °C for 30 min. The viable and dead cells are distinguished by their fluorescent color under the LSC microscope. PI passes cells via injured cell membranes of dead cells and interacts with nucleic acids, emitting red fluorescence. However, Hoechst 33342 cannot achieve to nucleic acids and it indicates blue color in cytosol of the neuronal cells. Images of bride field (black/white), blue and red cells were captured under the identical capture settings using the LSC microscope. ImageJ/Imaris software was used for analysis of neurons stained with PI and Hoechst. The samples were analyzed by the LSC microscopy fitted with a 20× objective. Live and death cell counts were expressed as %.

### Free Zn^2+^ assay by the LSC microscope

The SH-SY5Y neuronal cells were incubated with a 1 μM Zn^2+^ labeling fluorescent dye (RhodZin3-AM) with pluronic acid (0.1%) at 37 °C for 1 h. TPEN is a selective chelator of intracellular Zn^2+^ chelator and the cells were incubated with 100 nM TPEN at 37 °C for 1 h. The RhodZin3-AM and TPEN were removed by washing with HBS. The neuron suspension was dropped on a glass slide and covered with a rectangular coverslip. The images were captured using an inverted LSM800 LSC microscope with a ×40 oil objective as described in a previous study^45^. Image J software was used for analysis of fluorescent intensity. The mean fluorescence intensity as an arbitrary unit /neuron was used for expression.

### Western blotting

We used standard procedures for the Western blot analyses as described in previous studies^51^. The frozen SH-SY5Y samples were homogenized in lysis buffer; the supernatant was removed and conserved after centrifuge at 16,000×g, 20 min. Protein samples from the nerve samples were separated on SDS-PAGE gels and transferred to PVDF membranes. The blot was blocked for 1 h at room temperature with 5% nonfat dry milk in tris-buffered saline with 0.1% Tween 20. The membranes were incubated overnight at 4 °C with primary incubated with primary and secondary antibodies. Details of the primary and secondary antibody were given in our study^51^. Finally, they were visualized using an enhanced chemiluminescence system (Syngene G:Box Gel Imagination System, UK). Signal intensity was measured using ImageJ software for the analysis of PARP-1 expression levels. The data were normalized against β-actin protein. The data are presented as relative density over the pretreatment level.

### Assay of lipid peroxidation (MDA), reduced glutathione (rGSH) level and glutathione peroxidase (GPx) activity

The MDA and rGSH levels in SH-SY5Y neuronal cells (10^6^ cells in per ml) were spectrophotometrically (UV-1800, Shimadzu, Kyoto, Japan) measured at 532 and 412 nm by using the methods of Placer *et al*.^52^ and Sedlak and Lindsay^53^ as described in a previous study, respectively^21^. The rGSH level was expressed as μg/g protein For measuring GPx activity, a spectrophotometric method of Lawrence and Burk^54^ was used in the cells as described in previous studies^29^. The results of GPx was expressed as international unit (IU) of rGSH oxidized/min/g protein. The total protein in the supernatant was spectrophotometrically (Shimadzu UV-1800) assessed using Bradford reagent at 595 nm.

### Cytokine assays

The activities of IL-1β and TNF-α were assayed in the SH-SY5Y by using commercial the ELISA. The analyses were performed according to the manufacturer’s protocols (R&D Systems, Istanbul, Turkey) as described in a previous study^24^. Each cytokine sample was assayed in duplicates and the mean cytokine activity was calculated. The activities of IL-1β and TNF-α were indicated as ng/10^6^ cells.

### Quantitative real-time PCR

Total RNA was extracted from the frozen SH-SY5Y cells using a one-step method according to the instructions for TRIzol reagent (Invitrogen, USA). cDNA was then synthesized using reverse transcription in accordance with the kit instructions (Qiagen). The cell primer sequences were as follows: forward 5′-GAA GGA AAG AGG GGG TGT G-3′-19bp product, reverse 5′ CAT TGG TGA TGG CGT TGT AG-3′-20bp product). The TRPM2 mRNA expression level was performed in a RT-PCR (StepOnePlus, AB Applied Biosystem, Ankara, Turkey). RT-PCR amplification of cDNA was performed using a Quantitect SYBR Green RT-PCR Kit (Roche, USA). Relative gene expression was calculated using the 2^−∆∆CT^ method.

### Statistical analyses

All data were indicated as means ± standard deviation (SD). To assess the differences between treatment groups for each treatment, we used the one-way ANOVA. We used a post hoc test only when an ANOVA gave a statistically significant difference. We performed a Kruskal-Wallis in all data except Table 1 of the current study. We used a Student’s t test when comparing two groups (in the Table 1). The p ≤ 0.05 value was accepted statistically significant.

### Compliance with Ethical Standards

This article does not contain any studies with human and participants performed by any of the authors.

## Supplementary information

Supplementary Information.

## Data Availability

All methods in the manuscript were performed in accordance with the relevant guidelines and regulations of Suleyman Demirel University (Isparta, Turkey) by including a statement in the methods section to this effect. The dataset and analyses were generated in the BSN Health, Analyses, Innovation, Consultancy, Organization, Agriculture, Industry and Trade Limited Company, Göller Bölgesi Teknokenti, Isparta, Turkey and are available from the corresponding authors on reasonable request. Graphics and supplementary Fig. 3 in the manuscript were prepared by the corresponding author (Prof. Dr. Mustafa Nazıroğlu).

## References

[1] Martini, S., Austin, T., Aceti, A., Faldella, G. & Corvaglia, L. Free radicals and neonatal encephalopathy: mechanisms of injury, biomarkers, and antioxidant treatment perspectives. Pediatr Res. 10.1038/s41390-019-0639-6, [Epub ahead of print] (2019 Oct 26). 10.1038/s41390-019-0639-6 31655487

[2] Chen, S. D., Yang, J. L., Lin, T. K. & Yang, D. I. Emerging roles of sestrins in neurodegenerative diseases: Counteracting oxidative stress and beyond. *J Clin Med.* 8(7), 10.3390/jcm8071001 (2019). 10.3390/jcm8071001 PMC6678886 31324048

[3] Liu P (2016). Dihydromyricetin improves hypobaric hypoxia-induced memory impairment via modulation of SIRT3 signaling. Mol Neurobiol..

[4] Carrasco C, Nazıroǧlu M, Rodríguez AB, Pariente JA (2018). Neuropathic pain: Delving into the oxidative origin and the possible implication of transient receptor potential channels. Front Physiol..

[5] Liu X (2016). Resveratrol protects PC12 cells against OGD/ R-induced apoptosis via the mitochondrial-mediated signaling pathway. Acta Biochim Biophys Sin (Shanghai)..

[6] Gao J, Wang H, Li Y, Li W (2019). Resveratrol attenuates cerebral ischaemia reperfusion injury via modulating mitochondrial dynamics homeostasis and activating AMPK-Mfn1 pathway. Int J Exp Pathol..

[7] Guan P (2019). Resveratrol prevents chronic intermittent hypoxia-induced cardiac hypertrophy by targeting the PI3K/AKT/mTOR pathway. Life Sci..

[8] Wu X (2014). Resveratrol attenuates hypoxia/reoxygenation-induced Ca2+ overload by inhibiting the Wnt5a/Frizzled-2 pathway in rat H9c2 cells. Mol Med Rep..

[9] Kumar VS, Gopalakrishnan A, Nazıroğlu M, Rajanikant GK (2014). Calcium ion-the key player in cerebral ischemia. Curr Med Chem..

[10] Nazıroğlu M (2012). Molecular role of catalase on oxidative stress-induced Ca(2+) signaling and TRP cation channel activation in nervous system. J Recept Signal Transduct Res..

[11] Nazıroğlu M, Özgül C, Çelik Ö, Çiğ B, Sözbir E (2011). Aminoethoxydiphenyl borate and flufenamic acid inhibit Ca2+ influx through TRPM2 channels in rat dorsal root ganglion neurons activated by ADP-ribose and rotenone. J Membr Biol..

[12] Nazıroğlu M (2012). Melatonin modulates wireless (2.45 GHz)-induced oxidative injury through TRPM2 and voltage gated Ca(2+) channels in brain and dorsal root ganglion in rat. Physiol Behav..

[13] Öz A, Çelik Ö (2016). Curcumin inhibits oxidative stress-induced TRPM2 channel activation, calcium ion entry and apoptosis values in SH-SY5Y neuroblastoma cells: Involvement of transfection procedure. Mol Membr Biol..

[14] Sun Y (2018). TRPM2 promotes neurotoxin MPP(+)/MPTP-induced cell death. Mol Neurobiol..

[15] Nazıroğlu M, Braidy N (2017). Thermo-sensitive TRP channels: Novel targets for treating chemotherapy-induced peripheral pain. Front Physiol..

[16] Hatano N (2012). Hypoxia-inducible factor-1α (HIF1α) switches on transient receptor potential ankyrin repeat 1 (TRPA1) gene expression via a hypoxia response element-like motif to modulate cytokine release. J Biol Chem..

[17] Park J, Shim MK, Jin M, Rhyu MR, Lee Y (2016). Methyl syringate, a TRPA1 agonist represses hypoxia-induced cyclooxygenase-2 in lung cancer cells. Phytomedicine.

[18] Yang J (2018). Resveratrol treatment in different time-attenuated neuronal apoptosis after oxygen and glucose deprivation/reoxygenation via enhancing the activation of Nrf-2 signaling pathway *in vitro*. Cell Transplant..

[19] Zhang Q (2015). Resveratrol attenuates hypoxia-induced neurotoxicity through inhibiting microglial activation. Int Immunopharmacol..

[20] Perraud AL (2001). ADP-ribose gating of the calcium-permeable LTRPC2 channel revealed by Nudix motif homology. Nature.

[21] Park G, Jeong JW, Kim JE (2011). SIRT1 deficiency attenuates MPP+-induced apoptosis in dopaminergic cells. FEBS Lett..

[22] Said RS, Mantawy EM, El-Demerdash E (2019). Mechanistic perspective of protective effects of resveratrol against cisplatin-induced ovarian injury in rats: emphasis on anti-inflammatory and anti-apoptotic effects. Naunyn Schmiedebergs Arch Pharmacol..

[23] Li Q, Ma R, Zhang M (2018). CoCl(2) increases the expression of hypoxic markers HIF-1α, VEGF and CXCR4 in breast cancer MCF-7 cells. Oncol Lett..

[24] Öztürk Y, Günaydın C, Yalçın F, Nazıroğlu M, Braidy N (2019). Resveratrol enhances apoptotic and oxidant effects of paclitaxel through TRPM2 channel activation in DBTRG glioblastoma cells. Oxid Med Cell Longev..

[25] Joshi DC, Bakowska JC (2011). Determination of mitochondrial membrane potential and reactive oxygen species in live rat cortical neurons. J Vis Exp.

[26] Keil VC, Funke F, Zeug A, Schild D, Müller M (2011). Ratiometric high-resolution imaging of JC-1 fluorescence reveals the subcellular heterogeneity of astrocytic mitochondria. Pflugers Arch..

[27] Maret W (2019). The redox biology of redox-inert zinc ions. Free Radic Biol Med..

[28] Li X, Jiang LH (2018). Multiple molecular mechanisms form a positive feedback loop driving amyloid β42 peptide-induced neurotoxicity via activation of the TRPM2 channel in hippocampal neurons. Cell Death Dis..

[29] Nazıroğlu M, Lückhoff A (2008). A calcium influx pathway regulated separately by oxidative stress and ADP-Ribose in TRPM2 channels: single channel events. Neurochem Res..

[30] An X, *et al*. Increasing the TRPM2 channel expression in human neuroblastoma SH-SY5Y cells augments the susceptibility to ROS-induced cell death. Cells. 8(1). 10.3390/cells8010028 (2019). 10.3390/cells8010028 PMC6356620 30625984

[31] Miller BA (2013). The second member of transient receptor potential-melastatin channel family protects hearts from ischemia-reperfusion injury. Am J Physiol Heart Circ Physiol..

[32] Özgül C, Nazıroğlu M (2012). TRPM2 channel protective properties of N-acetylcysteine on cytosolic glutathione depletion dependent oxidative stress and Ca2+ influx in rat dorsal root ganglion. Physiol Behav..

[33] Belrose JC, Xie YF, Gierszewski LJ, MacDonald JF, Jackson MF (2012). Loss of glutathione homeostasis associated with neuronal senescence facilitates TRPM2 channel activation in cultured hippocampal pyramidal neurons. Mol Brain..

[34] Övey IS, Nazıroğlu M (2015). Homocysteine and cytosolic GSH depletion induce apoptosis and oxidative toxicity through cytosolic calcium overload in the hippocampus of aged mice: involvement of TRPM2 and TRPV1 channels. Neuroscience.

[35] Carrasco C, Rodríguez BA, Pariente JA (2015). Melatonin as a stabilizer of mitochondrial function: Role in diseases and aging. Turk J Biol..

[36] Ryan D, Drysdale AJ, Lafourcade C, Pertwee RG, Platt B (2009). Cannabidiol targets mitochondria to regulate intracellular Ca2+ levels. J Neurosci..

[37] Zhang S (2016). The Pivotal Role of Ca(2+) Homeostasis in PBDE-47-Induced Neuronal Apoptosis. Mol Neurobiol..

[38] Mai C (2020). TRPM2 channel: A novel target for alleviating ischaemia-reperfusion, chronic cerebral hypo-perfusion and neonatal hypoxic-ischaemic brain damage. J Cell Mol Med..

[39] Lin KL (2018). Resveratrol provides neuroprotective effects through modulation of mitochondrial dynamics and ERK1/2 regulated autophagy. Free Radic Res..

[40] Lin TK (2014). Resveratrol partially prevents rotenone-induced neurotoxicity in dopaminergic SH-SY5Y cells through induction of heme oxygenase-1 dependent autophagy. Int J Mol Sci..

[41] Gao Y (2018). Resveratrol mitigates the oxidative stress mediated by hypoxic-ischemic brain injury in neonatal rats via Nrf2/HO-1 pathway. Pharm Biol..

[42] Zhang Y (2018). DJ-1 preserving mitochondrial complex I activity plays a critical role in resveratrol-mediated cardioprotection against hypoxia/reoxygenation-induced oxidative stress. Biomed Pharmacother..

[43] Uğuz AC, Öz A, Nazıroğlu M (2016). Curcumin inhibits apoptosis by regulating intracellular calcium release, reactive oxygen species and mitochondrial depolarization levels in SH-SY5Y neuronal cells. J Recept Signal Transduct Res..

[44] Lin CH (2020). Neuroprotective effects of resveratrol against oxygen glucose deprivation induced mitochondrial dysfunction by activation of AMPK in SH-SY5Y cells with 3D gelatin scaffold. Brain Res..

[45] Nazıroğlu M (2019). Albumin evokes Ca(2+)-induced cell oxidative stress and apoptosis through TRPM2 channel in renal collecting duct cells reduced by curcumin. Sci Rep..

[46] Ismail AF, Salem AA, Eassawy MM (2016). Hepatoprotective effect of grape seed oil against carbon tetrachloride induced oxidative stress in liver of γ-irradiated rat. J Photochem Photobiol B..

[47] Chen SJ (2013). Role of TRPM2 in cell proliferation and susceptibility to oxidative stress. Am J Physiol Cell Physiol..

[48] Bao L (2016). Depletion of the human ion channel TRPM2 in neuroblastoma demonstrates its key role in cell survival through modulation of mitochondrial reactive oxygen species and bioenergetics. J Biol Chem..

[49] Gökçe Kütük S, Gökçe G, Kütük M, Gürses Cila HE, Nazıroğlu M (2019). Curcumin enhances cisplatin-induced human laryngeal squamous cancer cell death through activation of TRPM2 channel and mitochondrial oxidative stress. Sci Rep..

[50] McHugh D, Flemming R, Xu SZ, Perraud AL, Beech DJ (2003). Critical intracellular Ca2+ dependence of transient receptor potential melastatin 2 (TRPM2) cation channel activation. J Biol Chem..

[51] Yazğan Y, Nazıroğlu M (2017). Ovariectomy-induced mitochondrial oxidative stress, apoptosis, and calcium ion influx through TRPA1, TRPM2, and TRPV1 are prevented by 17β-estradiol, tamoxifen, and raloxifene in the hippocampus and dorsal root ganglion of rats. Mol Neurobiol..

[52] Placer ZA, Cushman L, Johnson BC (1966). Estimation of products of lipid peroxidation (malonyl dialdehyde) in biological fluids. Anal Biochem.

[53] Sedlak J, Lindsay RHC (1968). Estimation of total, protein bound and non-protein sulfhydryl groups in tissue with Ellmann’ s reagent. Anal Biochem..

[54] Lawrence RA, Burk RF (2012). Glutathione peroxidase activity in selenium-deficient rat liver. Biochem Biophys Res Commun..

