# Supplementary material for: Resveratrol attenuates hypoxia-induced neuronal cell death, inflammation and mitochondrial oxidative stress by modulation of TRPM2 channel
Source: Sci Rep. 2020 Apr 15;10:6449. doi: 10.1038/s41598-020-63577-5 (PMC7160154; doi:10.1038/s41598-020-63577-5)

**Resveratrol attenuates hypoxia-induced neuronal cell death, inflammation and mitochondrial oxidative stress by modulation of TRPM2 channel**

**Yener Akyuva^1^, Mustafa Nazıroğlu^2,3^**

^1^Departmant of Neurosurgery, Faculty of Medicine, Hatay Mustafa Kemal University, Hatay, Turkey

^2^Director of Neuroscience Research Center (NOROBAM), Suleyman Demirel University, Isparta, Turkey

^3^Drug Discovery Unit, BSN Health, Analysis and Innovation Ltd. Inc. Teknokent, Isparta, Turkey

**Supplementary Fig. 1**. Full length Western blots of PARP-1 and β-actin in SH-SY5Y neuronal cells for Figure 7c (n=3).


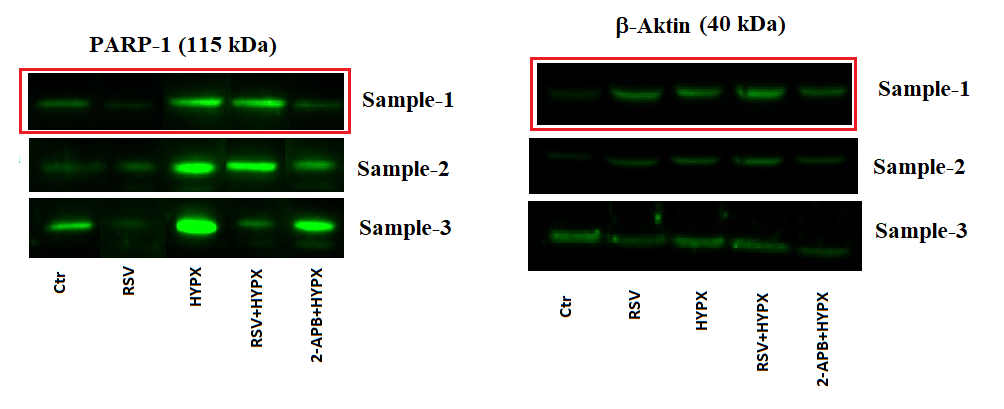


**Supplementary Fig. 2**. **Resveratrol (RSV and 50 μM) and ACA (25 μM) diminished hypoxia (CoCl_2_ and 50 μM)-induced increase of TRPM2 mRNA expression level, IL-1β and TNF-α activities in the SH-SY5Y cells. (n=4 and mean ± SD).** The TRPM2 mRNA expression level was analyzed by using the RT-PCR, although IL-1β and TNF-α activities were assayed by using the ELISA. **a.** The mean values of TRPM2 mRNA expression level. **b and c.** The mean activities of IL-1β and TNF-α respectively. (^*^p ≤ 0.001 versus Ctr and RSV groups. ^**^p ≤ 0.001 versus HYPX group).


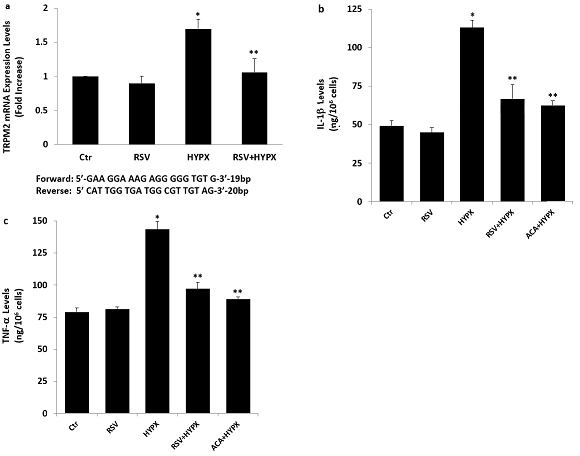


**Supplementary Fig. 3**. **Modulator actions of resveratrol on the TRPM2‐dependent molecular mechanisms in the delaying neuronal death.** Increased production of reactive oxygen species (ROS) during hypoxia and subsequent activation of the TRPM2 channel in SH-SY5Y neuronal cells induce down‐regulation of the GSH and GSH‐mediated survival redox signaling pathway and up‐regulation of the caspase -3 and -9‐mediated apoptosis‐promoting signaling molecular pathways, resulting in neuronal cell death. ROS also induces activation of the TRPM2 channel in the mitochondria as well as on the cell surface via promoting ADPR generation catalyzed by poly(ADPR) polymerase-1 (PARP-1) and poly(ADPR) glycohydrolase (PARG) in the nucleus. Activation of the TRPM2 channel in the mitochondria increases mitochondrial uptake of Zn^2+^ that triggers mitochondrial loss and dysfunction and mitochondrial ROS generation. Therefore, inhibition of the TRPM2 channel by resveratrol, ACA, 2-APB treatments sets in motion a positive feedback mechanism ultimately modulates mitochondrial dysfunction and neuronal death


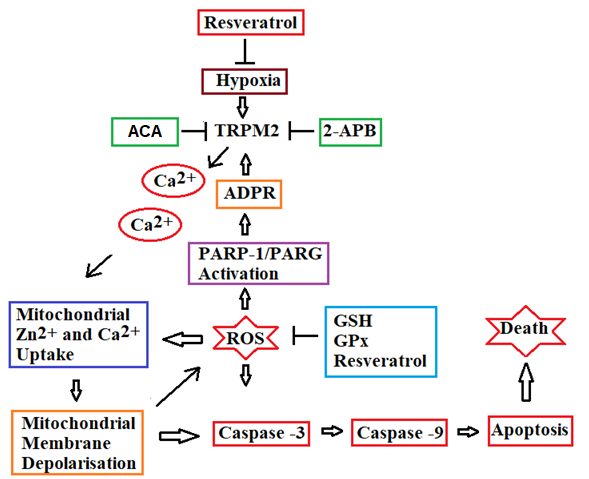

Supplement: Supplementary file 1 — Supplementary Information. [file 41598_2020_63577_MOESM1_ESM.docx]
